# Reduced Global-Brain Functional Connectivity of the Cerebello-Thalamo-Cortical Network in Patients With Dry Eye Disease

**DOI:** 10.3389/fnhum.2020.572693

**Published:** 2020-09-25

**Authors:** Pan Pan, Shubao Wei, Yangpan Ou, Feng Liu, Huabing Li, Wenyan Jiang, Wenmei Li, Yiwu Lei, Wenbin Guo, Shuguang Luo

**Affiliations:** ^1^National Clinical Research Center for Mental Disorders, and Department of Psychiatry, The Second Xiangya Hospital of Central South University, Changsha, China; ^2^Department of Neurology, The First Affiliated Hospital of Guangxi Medical University, Nanning, China; ^3^Department of Radiology, Tianjin Medical University General Hospital, Tianjin, China; ^4^Department of Radiology, The Second Xiangya Hospital of Central South University, Changsha, China; ^5^Department of Radiology, The First Affiliated Hospital of Guangxi Medical University, Nanning, China; ^6^The Third People's Hospital of Foshan, Foshan, China

**Keywords:** dry eye disease, global-brain functional connectivity, network, functional magnetic resonance imaging, support vector machine

## Abstract

**Background:** The pathophysiology of patients with dry eye disease (DED) is associated with abnormal functional connectivity (FC). The present study aims to probe alterations of voxel-wise brain-wide FC in patient with DED at rest in an unbiased way.

**Method:** A total of 20 patients with DED and 23 controls matched by age, sex, and years of education underwent resting-state functional magnetic resonance imaging scans. Global-brain FC (GFC) was adopted to analyze the images. Support vector machine (SVM) was utilized to differentiate the patients from the controls.

**Results:** Compared with the controls, patients with DED exhibited decreased GFC in the right cerebellum lobule VIII/inferior semi-lunar lobule and left thalamus that belonged to the cerebello-thalamo-cortical network. The GFC values in the left thalamus were positively correlated to the illness duration (*r* = 0.589, *p* = 0.006) in the patients. Decreased GFC values in the left thalamus could be used to discriminate the patients from the controls with optimal accuracy, sensitivity and specificity (88.37, 85.00, and 91.30%).

**Conclusions:** Our findings indicate that decreased GFC in the brain regions associated with cerebello-thalamo-cortical network may provide a new insight for understanding the pathological changes of FC in DED. GFC values in the left thalamus may be utilized as a potential biomarker to identify the patients from the controls.

## Introduction

The surface of the eyes is covered with a layer of tears. A stable tear film provides a comfortable environment to the eyes and serves as a good refractive media of the ocular surface (cornea, conjunctiva, accessory lacrimal gland and meibomian gland). Lack of tear membrane components will break the stability of the tear film resulting in ocular surface drying and the conjunctive epithelium damaged by dehydration, known as dry eye syndrome (DED) (Pflugfelder et al., 1999, 2018; Brewitt and Sistani, 2001). DED, one of the most common ocular surface diseases, caused by a variety of factors, is characterized by the absence of mucous components in tears, abnormal distribution of tears on the ocular surface, and increased evaporation of tears (Tabbara and Sharara, 1998). The main clinical manifestations of DED include painful ocular symptoms often described as “burning and painful” (Lemp, 2007; Kalangara et al., 2017; Galor et al., 2018), visual impairment and unstable tear film with potential ocular surface damage, accompanied by increased tear osmotic pressure and ocular surface inflammation (Lemp, 2007). The aggravation of symptoms will lead to a range of serious eye disease including corneal abrasion, filamentous keratitis, and corneal ulcers eventually leading to nubecula and vision loss. However, the pathophysiology of DED remains unclear at present.

In recent years, “neurosensory abnormalities” have been added to the definition of DED (Craig et al., 2017). A growing literature suggested that dry eye symptoms in patients with DED should be better conceptualized as neuropathic eye pain, a manifestation of a central pain processing disorder (Galor et al., 2015; Kalangara et al., 2017; Levitt et al., 2017). Changes in surface microenvironment have taken place to control the secretion of ocular surface glands and blink activity through the ocular sensory pathway. Persistent changes will lead to ocular surface dyskinesia and neuropathic pain caused by functional dysfunction (Belmonte et al., 2017). Dysfunction is a medical condition that normal function of the body is impaired by autonomic dysfunction caused by cortical dysfunction (Liang et al., 2019). Previous studies showed that the occurrence of ocular surface pain is associated with tears deficiency, and repeated ocular sensory nerve injury is involved in the persistence of pain leading to transition from acute pain to chronic pain (Kalangara et al., 2017; Levitt et al., 2017). Sustained structural and functional changes in ocular sensory pathway could cause neuropathic pain and hypotonia of the ocular surface in patients with DED (Belmonte et al., 2017). Functional magnetic resonance imaging (fMRI) has been gradually applied in the clinical researches of DED. Research suggests that chronic peripheral nerve damage will lead to pathological neuroplasticity in the central nervous system (CNS) resulting in reduced nociceptor excitatory thresholds (Levitt et al., 2017). Previous researches showed that patients with DED had extensive alterations in brain function, and abnormal brain function played an important role in the maintenance and development of dry eye symptoms (Rahman et al., 2015; Levitt et al., 2017). fMRI has been used to explore abnormal functional activity in eye diseases such as glaucoma, amblyopia and corneal ulcers (Lin et al., 2012; Li et al., 2014; Rahman et al., 2015; Chen et al., 2017; Wang et al., 2017; Xu et al., 2019). However, it remains unclear whether patients with DED have abnormal GFC in certain brain regions.

To further clarify the pathophysiology of DED, a voxel-wise global-brain functional connectivity (GFC) approach was used to investigate the differences of functional organization between patients with DED and healthy controls. GFC is helpful to obtain voxel-wise whole-brain functional connectivity (FC) in an unbiased way (Li et al., 2016; Cui et al., 2018). Previous researches have demonstrated that GFC is a powerful and replicable data-driven analysis capable of identifying major intrinsic networks (Cole et al., 2009; Murrough et al., 2016). GFC provides an approach to measure the connectivity of all voxels in the brain relative to all other voxels by using a metric that does not require prior selection of seeds or networks (Meier et al., 2016; Zhurakovskaya et al., 2016; Cui et al., 2018; Pan et al., 2019a). Several researches of brain mechanisms have focused on the functional connectivity (FC) between preselected brain regions by using a region of interest (ROI) method (Pires et al., 2012; Meijer and Goraj, 2014; Giorgio and De Stefano, 2016; Lee et al., 2018). This approach is insufficient although the results from ROI are informative. Different ROI selection may yield different results due to potentially biased results based on preselected ROIs. In addition, it may not cover important brain regions associated with the core pathological changes in DED. By contrast, the novel aspect of the present study is that FC abnormalities in patients with DED were examined in a voxel-wise brain-wide way. GFC was considered as a suitable method to examine the differences in a large-scale functional organization in the brain for providing an unbiased way to measure brain function. Therefore, the purpose of the GFC method adopted in the study was to observe the mechanism of brain from the perspective of FC alterations across the whole brain. The potential FC alterations in patients with DED may be detected by this method. Finally, we used the receiver operating characteristic curve (ROC) and support vector machine (SVM) methods to investigate whether abnormal GFC in relevant brain areas could be considered as potential image biomarkers to discriminate patients from controls with good sensitivity and specificity.

## Materials and Methods

### Subjects

A total of 20 right-handed patients with DED were recruited from the Frist Affiliated Hospital of Guangxi Medical University. DED was diagnosed according to DED diagnostic guidelines published by the Dry Eye Workshop in 2007 (Lemp, 2007). A total of 23 right-handed healthy controls without symptoms of neurological and ophthalmic disease were recruited from the local community at the same time. All participants aged from 18 to 65 years old. Most symptoms did not differ across different ages. However, burning sensation was common in patients aged 18~45 years old, whereas photophobia was common in patients aged 46~65 years old (Gao et al., 2011). Healthy controls were group-matched with the patients in terms of age, sex ratio, and years of education.

The exclusion criteria for patients with DED were as follows: (1) any history of connective tissue disease, such as rheumatoid arthritis and systemic lupus erythematosus; (2) any history hypertensive encephalopathy, metabolic encephalopathy, and lesions of the CNS caused by infection or other reasons. Healthy controls shared the following exclusion criteria: (1) any history of severe neurological diseases or ophthalmic diseases; (2) any history of serious surgery of internal medicine diseases; and (3) any family history of serious neuropsychiatric disorders or ophthalmic diseases in their first-degree relatives. The participants who did not meet the standard for MRI or showed alterations under conventional MRI scans were also excluded.

The study was approved by the Local Ethics Committee of the First Affiliated Hospital of Guangxi Medical University. All procedures performed in the studies involving human participants were in accordance with the ethical standards of the institutional or national research committee and with the 1964 Helsinki declaration and its later amendments or comparable ethical standards. All participants were provided with a written informed consent before the experiment.

### MRI Parameters

MRI images were captured by a Siemens 3.0T scanner. All participants were required to remain motionless and awake with their eyes closed. Participants were given soft earplugs and foam pads to reduce scanning noise and head motion. Resting-state functional images of slice-order type were obtained by an ascend type using the following parameters: repetition time/echo time = 2,000 ms/30 ms, inversion time = 900 ms, 30 slices, 64 × 64 matrix, 90° flip angle, 240 mm field of view, 4 mm slice thickness, 0.4 mm gap, and 250 volumes lasting for 500 s. After the scan, participants were asked some questions to confirm the wakefulness during the scan. For example, whether the participant fell asleep or the sound frequency of the machine changed during the scanning.

### fMRI Data Analysis

Functional images were preprocessed automatically by using the DPABI software (Yan et al., 2016). Because there was time for participants to adapt to the scan environment, the first 10 volumes were removed to ensure a steady-state condition. We excluded the participants whose head movement was more than 2 mm of translation or 2° of rotation in any directions. The time series of fMRI were first corrected for within-scan acquisition time difference between slices and head motion. All realigned images were spatially normalized to the Montreal Neurological Institute (MNI) EPI space in SPM8 and resampled to 3 × 3 × 3 mm^3^ (Liu et al., 2015). After normalization, the images were smoothed with 4 mm full width on the half maximum Gaussian kernel. Due to the limitation of imaging methods, the lesions of fMRI images were often hid in noise signals which might cause missed diagnosis and even misdiagnosis. The features of the lesion site would be more obvious after proper noise reduction pretreatment. The most common method of noise reduction is smooth. The smooth method is also used in previous studies on ophthalmic diseases (Chen et al., 2017; Wang et al., 2017; Xu et al., 2019). The time series were further linearly detrended and temporally band-pass filtered (0.01–0.08 Hz). Finally, several covariates were removed including Friston-24 head motion parameters obtained by rigid body correction, signal from white-matter centered region, and signal from cerebrospinal fluid. As described in a previous study, the global signal was not removed (Hahamy et al., 2014). The frame displacement (FD) value of each subject was calculated based on a previous study (Power et al., 2012). Scrubbing that removing time point with FD > 0.2 mm was used to control the effect of head motion.

### GFC Analysis

The GFC method used in the study is similar to that used in our previous studies (Cui et al., 2018, 2020; Ding et al., 2019; Pan et al., 2019a,b; Li et al., 2020). GFC is a measure of functional connectivity of all voxels in the brain with respect to other voxels. For each participant, we calculated the average values of correlations between each voxel's time series and every other voxel in the gray matter of the whole brain in MATLAB, which was defined as GFC of this voxel. The threshold setting classified voxel with probability of >0.2 as gray matter, and the gray matter mask would be produced by the gray matter probability map in SPM8 (Liu et al., 2015). The GFC evaluation was defined as,

$$\text{GFC}=\sum_{b=1}^{n}\frac{r\left(T_{a},T_{b}\right)}{n-1}$$

The process of GFC involves calculating the Pearson correlation coefficients (r) between a given voxel's time series and all other voxels' time series, converting all correlations into Fisher z-scores matrix (Wang et al., 2015; Cui et al., 2018; Pan et al., 2019a,b; Li et al., 2020). The process generates a map for each participant, where each voxel value represents the average connectivity of the voxel with the rest of the brain. The GFC maps were generated by combining GFC of all voxels.

Two-sample *t*-tests were conducted on the GFC maps between patients with DED and healthy controls. The mean FD and age were used as covariates of no interest. The significance level was set as *p* < 0.05 by using the family wise error (FWE) correction method.

### Correlation Analysis

Mean *z* values of the brain clusters with abnormal GFC were extracted. Pearson correlation analyses were performed to explore the relationship between abnormal GFC values and illness duration in the DED group after the normality of the data being checked. In the present study, we tested the independent hypotheses on the same data at the significance level of *p* < 0.05 through an approach of Bonferroni which was the strictest multiple test correction method.

### Classification Analysis by Using SVM

As a good tool of classification especially suitable for the case of small samples (Vapnik, 1995), SVM was utilized to examine whether abnormal GFC values in brain areas could be applied to distinguish patients from controls (Chih-Chung and Chih-Jen, 2011) using the LIBSVM software package in the MATLAB. The type of kernel was the default Gaussian kernel in the LIBSVM. The classification performance of the unobserved data was evaluated by dividing the sample set into a training set and a test set. A random SVM cluster was constructed for classification and feature selection based on the brain fMRI data of the subjects. The LIBSVM adopted a “leave-one-out” method that was cross-validated to optimize parameters and obtain satisfactory sensitivity and specificity (Liu et al., 2015; Yan et al., 2016).

The receiver operating characteristic curve (ROC) analysis was used to validate the SVM results, once significant differences in GFC values were observed between the patients and the controls.

## Results

### Characteristics of the Subjects

The sample of the present study included 20 patients with DED and 23 healthy controls. Continuous variables including age, years of education and FD were analyzed with two-sample *t*-tests. A Chi-squared test was utilized for sex distribution. Compared with the control group, age (*p* = 0.23), sex ration (*p* = 0.19), FD (*p* = 0.23), and years of education (*p* = 0.08) of the patient group were not statistically significant. The detailed information of demographic and clinical characteristics of the participants were showed in Table 1.

**Table 1:** Characteristics of the participants.

	**Patients (*n* = 20)**	**Controls (*n* = 23)**	***p*-value**
Sex (male/female)	7/13	4/19	0.19^a^
Age (years)	52.55 ± 8.66	49.69 ± 6.51	0.23^b^
Education (years)	10.20 ± 3.56	8.61 ± 2.27	0.08^b^
FD (mm)	0.31 ± 0.20	0.39 ± 0.26	0.23^b^
Illness duration (months)	20.75 ± 15.37		

a *The p-value for sex distribution was obtained by a chi-square test*.

b *The p-values were obtained by independent-samples t-tests*.

*FD, Framewise displacement*.

### Group Differences in GFC

**Figure 1:**
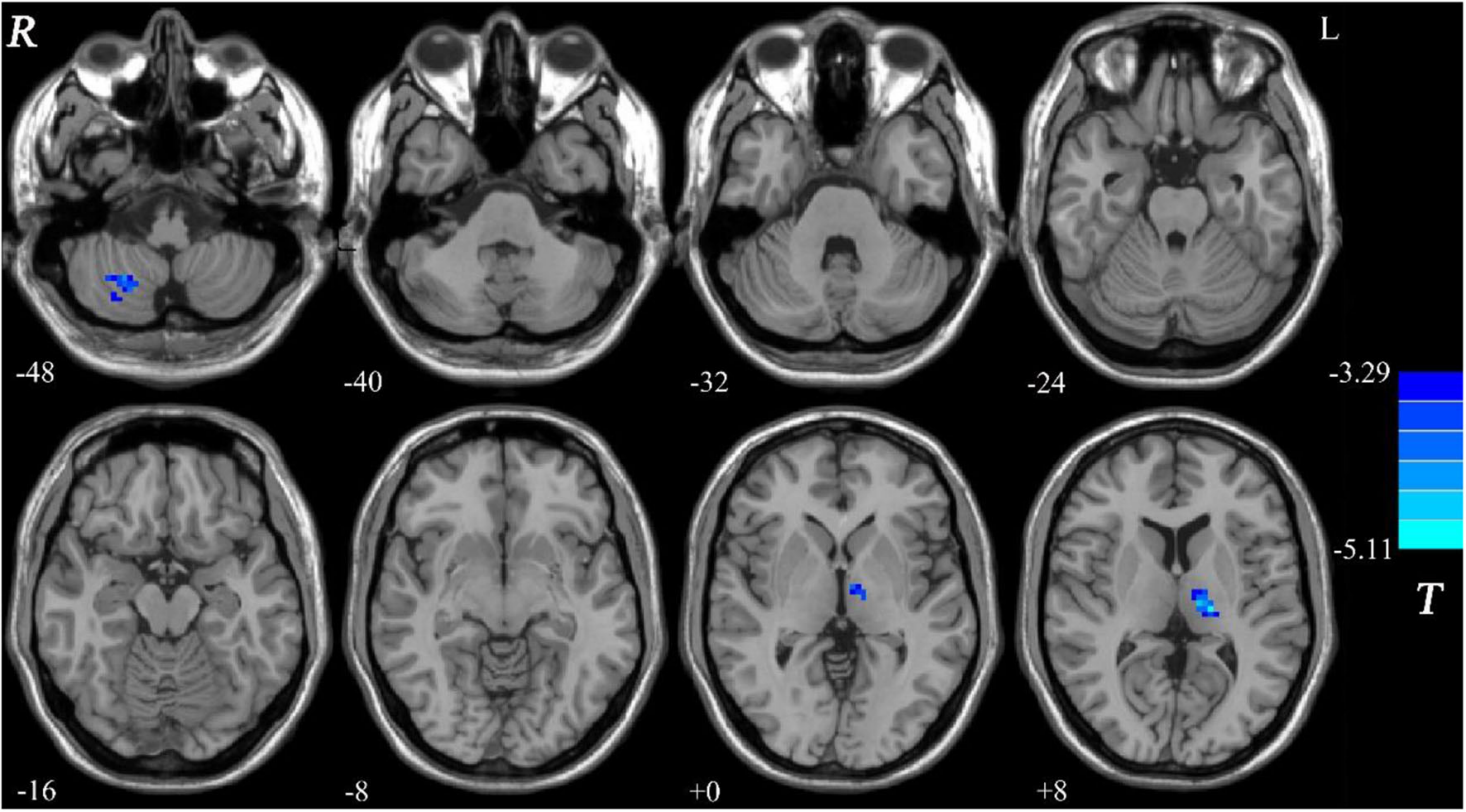
Decreased GFC in patients with DED relative to healthy controls. GFC, global-brain functional connectivity; DED, dry eye disease.

Compared with the controls, patients with DED exhibited decreased GFC in the right cerebellum lobule VIII/inferior semi-lunar lobule and left thalamus (Figure 1 and Table 2). No brain region exhibited increased GFC in the patients relative to the controls.

**Table 2:** Regions with decreased GFC in the patients.

**Cluster location**	**Peak (MNI)**			**Number of voxels**	***T* value^**a**^**
	**x**	**y**	**z**		
Right cerebellum lobule VIII/inferior semi-lunar lobule	24	−60	−48	32	−4.3311
Left thalamus	−18	−21	9	67	−4.1686

*GFC, global-brain functional connectivity; MNI, Montreal Neurological Institute*.

a *A negative T value represents decreased GFC in the patients relative to the controls*.

### Correlations Between GFC and Clinical Variable

**Figure 2:**
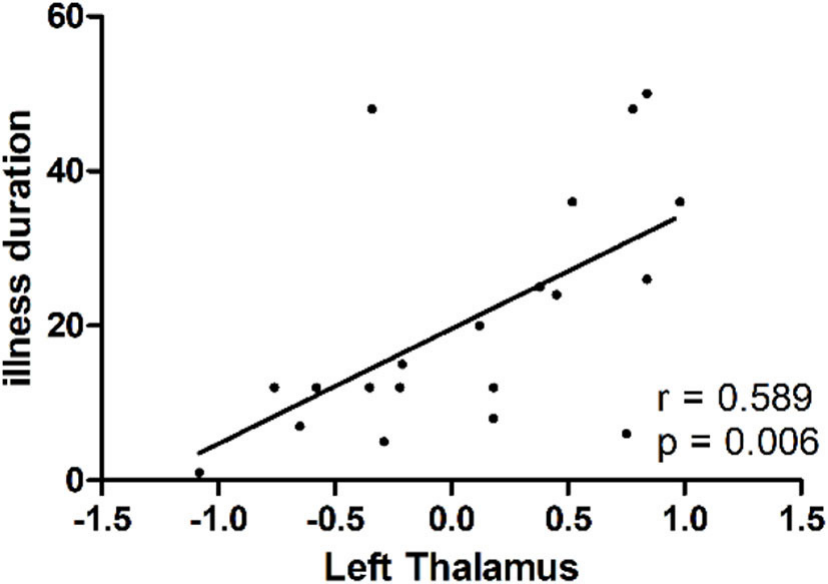
A positive correlation (*r* = 0.589, *p* = 0.006) between GFC values in the left thalamus and illness duration in patients with dry eye disease. GFC, global-brain functional connectivity.

As shown in Figure 2, a positive correlation was observed between GFC values in the left thalamus and illness duration in the patients (*r* = 0.589, *p* = 0.006).

### Distinguishing Patients With DED From Controls

**Figure 3:**
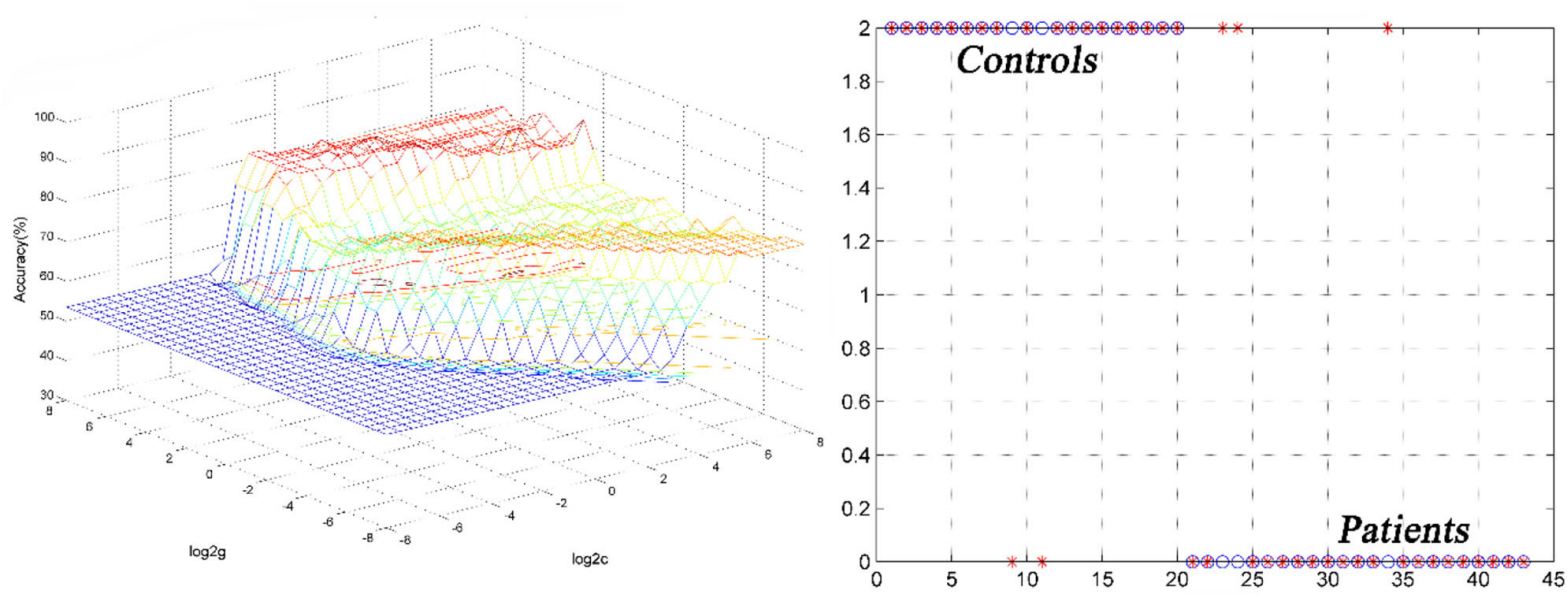
3D view of classified accuracy with best parameters using the GFC values in the left thalamus to differentiate the patients from the controls. The result was obtained in the LIBSVM using a “leave-one-out” approach with default Gaussian kernel. GFC, global-brain functional connectivity.

SVM analysis was utilized to determine whether abnormal GFC of these brain regions could distinguish patients with DED from healthy controls. Decreased GFC in the left thalamus exhibited the highest accuracy (88.37%), sensitivity (85.00%) and specificity (91.30%) that could be applied to identify the patients from the controls (Figure 3). As shown in Figure 3 and Table 3, the accuracy of another brain region was unsatisfactory.

**Table 3:** Differentiate the patients from the controls by GFC values in each brain region with the SVM method.

**Brain regions**	**Accuracy**	**Sensitivity**	**Specificity**
Right cerebellum lobule VIII/inferior semi-lunar lobule	76.74% (33/43)	75.00% (15/20)	78.26% (18/23)
Left thalamus	88.37% (38/43)	85.00% (17/20)	91.30% (21/23)

*GFC, global-brain functional connectivity; SVM, support vector machines*.

**Figure 4:**
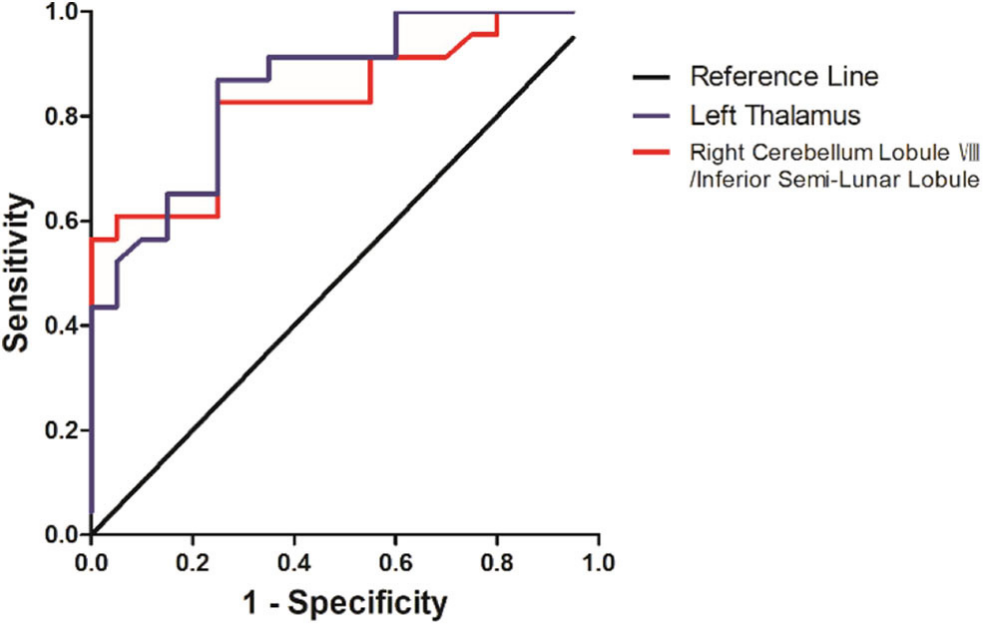
Differentiating the patients from the controls with the receiver operating characteristic curve method by using the GFC values in the left thalamus and right cerebellum lobule VIII/inferior semi-lunar.

The SVM results were further validated by the ROC method. The results exhibited that the GFC values in the left thalamus could be applied to identify patients from controls with optimal specificity (95.00%) and sensitivity (78.26%) (Figure 4 and Table 4).

**Table 4:** ROC analyses for differentiating the patients from the controls by using the GFC values.

**Brain regions**	**Area under the curve**	**Cut-off point**	**Sensitivity**	**Specificity**
Left thalamus	0.859	−0.3377	95.00% (19/20)	78.26% (18/23)
Right cerebellum lobule VIII/inferior semi-lunar lobule	0.830	0.0586	90.00% (18/20)	56.52% (13/23)

*ROC, receiver operating characteristic curve; GFC, global-brain functional connectivity*.

## Discussion

Compared with healthy controls, patients with DED exhibited significantly decreased GFC values in the cerebello-thalamo-cortical network including the thalamus and cerebellar lobule. Furthermore, GFC values in the left thalamus were positivity correlated with illness duration in the patients. GFC values in the left thalamus could correctly distinguish patients from healthy controls with optimal accuracy, sensitivity, and specificity.

Previous animal neurophysiological studies (Optican and Robinson, 1981; Barash et al., 1999) and human neurotrauma psychology studies (Waespe and Baumgartner, 1992; Panouillères et al., 2013) provided clear evidence that the cerebellum acted as an important role in the saccade adaptation. Alteration in the cerebellum lobule activity was associated with saccade adaptation (Guillaume et al., 2018). Spontaneous nystagmus might affect visual function in patients with unilateral cerebellar lobules and inferior semi-lunar lobule injury (Lee and Kim, 2020). The cerebellar lobule plays an important role in different coding of visual spatial cognition and visual working memory. The cerebellum lobule VIII was the most significantly activated area during above process (Sobczak-Edmans et al., 2016). A previous study observed functional separation between spatial coding and visual working memory processing in the cerebellum lobule (Brissenden et al., 2018). To sum up, cerebellum lobule may participate in the formation of visual working memory. The occurrence of visual impairment in patient with DED may be related to decreased GFC in the right cerebellum lobule VIII/inferior semi-lunar lobule.

The thalamus, located bilaterally around the third ventricle, is the largest elliptic gray matter mixed nucleus mass in the diencephalon. The main function of the thalamus as a relay station for the cerebral cortex to transmit damage information is to synthesize and distribute the sensory to different brain areas (Ab Aziz and Ahmad, 2006; Yen and Lu, 2013). Previous studies showed that the thalamus plays an important role in acute or chronic aching, anesthesia, and analgesia. The thalamus was often activated in fMRI experiments with pain (Heinke and Schwarzbauer, 2002; Martuzzi et al., 2010; Mhuircheartaigh et al., 2010). The descending inhibitory system composed of the thalamus and dorsal root ganglion transmitted aching information to the sensory cortex, and responded to aching during the transmission process and then gave feedback to external traumatic stimuli (Ab Aziz and Ahmad, 2006). Significantly decreased GFC in the left thalamus was observed in patients with DED in this study. Decreased GFC in the thalamus might lead to repeated nerve injury, which changed the stress activated state into the continuously activate state, and then made the pain threshold from enhancement to reduced feedback during the transformation of ocular pain from acute phase to persistent pain in patient with DED. Decreased GFC in the thalamus may be one cause of persistent eye aching in patients with DED. Notably, decreased GFC in the left thalamus was positivity correlated with illness duration in the patients. Abnormal GFC in the left thalamus may be affected the duration of the disease in patients with DED and provides theoretical basis for the pathophysiological of course of the disease in DED.

Specific interconnections exist widely between the thalamus and the cerebral cortex. The thalamic nuclei were connected with the corresponding cerebral cortex to form the thalamus-sensory projection system through these connections, and received and transmitted sensory information in this way (Klingner et al., 2013; Cheng et al., 2015; Penner et al., 2016). The cerebello-thalamo-cortical network was a complete functional network that could carry out visual information and pain information transmission, processing and response in both internal and external of the visual brain region. GFC alteration in the cerebello-thalamo-cortical network may be associated with visual impairment and persistent pain symptoms in patients with DED.

Abnormal GFC values might be utilized as potential biomarkers to identify patients from healthy controls. SVM analyses were conducted to determine whether the GFC values in the right cerebellum lobule VIII/inferior semi-lunar lobule and left thalamus could differentiate the patients from the controls with good sensitivities, specificities, and accuracies. Sensitivity or specificity higher than 0.75 indicated that decreased GFC in the left thalamus to go an accurate indicators (Gong et al., 2011). However, specificity < 0.6 for decreased GFC in right cerebellum lobule VIII/inferior semi-lunar lobule seems to be an indicator with poor accuracy. The accuracy, sensitivity and specificity of GFC values in the left thalamus were more than 0.8 (Table 3), which were optimal for the established diagnostic indicators (Swets, 1988). The ROC results were used to validate the SVM results, which showed that the area under the curve of the left thalamus was more than 0.7, an acceptable accuracy for established diagnostic indicators. Hence, we suggested that decreased GFC in the left thalamus could serve as a potential biomarker to discriminate the patients with DED from the controls.

Several limitations should be considered in the present study. First, the data size is small, the methods and results need to be validated on a larger data set. Second, clinical characteristics such as the data on age of onset were collected retrospectively which might have limited the accuracy of the information. Further assessment of other non-sensory manifestation could be used to provide a comprehensive explanation in patients with DED. Finally, the MNI template used in the study came from a Caucasian population which might bias the present findings from Chinese subjects.

## Conclusion

Despite the limitation, the present study indicates that decreased GFC exists in brain regions of the cerebello-thalamo-cortical network in patients with DED. Decreased GFC values in the left thalamus may be utilized as a potential biomarker to differentiate the patients from the controls. Thus, this study provides new insights into the pathological changes of GFC in DED.

## Data Availability Statement

All datasets generated for this study are included in the article/supplementary material.

## Ethics Statement

The studies involving human participants were reviewed and approved by Local Ethics Committee of the First Affiliated Hospital of Guangxi Medical University. The patients/participants provided their written informed consent to participate in this study.

## Author Contributions

WG and SL provided the conception of the work. SW, PP, YO, and WJ collected the data. FL, HL, WL, and YL were responsible for data analysis and interpretation. The manuscript was drafted by PP and critically revised by WG. All authors have given approval to final version of the manuscript. The manuscript was written through contributions of all authors.

## Conflict of Interest

The authors declare that the research was conducted in the absence of any commercial or financial relationships that could be construed as a potential conflict of interest.
